# Adult Refsum Disease in Puerto Rico: A Case Report

**DOI:** 10.7759/cureus.45426

**Published:** 2023-09-17

**Authors:** Raúl Y Ramos-Sánchez, José J López-Fontanet, Natalio Izquierdo

**Affiliations:** 1 Ophthalmology, University of Puerto Rico, Medical Sciences Campus, San Juan, PRI

**Keywords:** peripheral sensory neuropathy, retinitis pigmentosa, deafness, anosmia, refsum disease

## Abstract

Patients with adult Refsum Disease (ARD) have retinitis pigmentosa and thus nyctalopia, anosmia, sensorineural deafness, polyneuropathy, and ataxia. Upon physical examination, patients with ARD have congenital short metacarpals, metatarsals, and cardiac arrhythmias. Manifestations due to the lack of phytanoyl-CoA hydroxylase in peroxisomes needed for alpha-oxidation of phytanic acid lead patients to accumulate phytanic acid in their body tissues. To our knowledge, no consensus for clinical diagnostic criteria for patients with ARD has been published.

Our patient had nyctalopia, retinal findings, and visual field results compatible with retinitis pigmentosa. Additionally, the patient had decreased macular thickness and volume in both eyes, the findings being worse in the left eye. The patient had undergone hand surgery due to chronic pain in both hands, as well as his fourth and fifth metatarsal bones were shortened. Interestingly, audiology evaluation showed mild hearing loss in the right ear and mild to moderate hearing loss in the left ear.

Inheritance patterns in patients with ARD have been described. Physical examination, phytanic acid evaluation, and genetic studies may all help reach an ARD diagnosis. This is the first report of adult Refsum disease in Puerto Rico.

## Introduction

Sir Sigvald Refsum first described adult Refsum Disease (ARD) in 1945 [1]. ARD is a rare recessively inherited metabolic disease affecting phytanic acid metabolism. It causes retinitis pigmentosa and thus nyctalopia [2]; anosmia [3]; sensorineural deafness, polyneuropathy, and ataxia [4], amongst other clinical signs. By limiting dietary intake, plasma phytanic acid levels fall with an improvement in the neurological signs. The onset of retinitis pigmentosa usually precedes biochemical diagnosis by several years by which time the retinal damage is severe. Patients with the syndrome develop symptoms and clinical manifestations during late childhood or early adulthood.

Upon physical examination, patients with ARD have congenital short metacarpals and metatarsals [5], and cardiac arrhythmias [6]. To our knowledge, no consensus for clinical diagnostic criteria for patients with ARD has been published.

Phytanic acid is a fatty acid with methyl side chains and cannot be catabolized directly by the beta-oxidation process used by other fatty acids because of the steric hindrance of the methyl side chain at position 3. It, therefore, depends on an initial alpha-oxidation and decarboxylation before beta-oxidation can metabolize the rest of the chain. By using other radio-labeled beta-methyl fatty acids Stokke et al. [7] found that the initial alpha-oxidation and decarboxylation were affected in Refsum disease. Fatty acid catabolism can also be initiated from the non-carboxyl end of the chain by omega-oxidation.

In Refsum disease, the above-mentioned process becomes the only metabolic pathway for phytanic acid. The capacity of this pathway is limited to only 10 mg of phytanic acid a day and may vary between individuals. The average Western diet contains 50 mg of phytanic acid, derived largely from dairy and ruminant fats. Although unbound phytol can be metabolized to phytanic acid, green vegetables are bound to chlorophyll so it is not easily absorbed in man. Therefore, in Refsum disease phytanic acid accumulates in the body and is thought to lead to the clinical manifestations of this disease [8]. For this reason, phytanic acid may be measured in serum.

ARD is a very rare disease without racial associations. It affects both sexes equally. ARD is inherited as an autosomal recessive disease. Identification of biallelic pathogenic variants in either *PHYH *or *PEX7* genes [9] on molecular genetic testing and comprehensive genomic testing may all clarify the genetic basis of the disease.

Mutations on the *PHYH* gene account for approximately 90% of cases [8]. This gene is located at chromosome 10 (p11.2-pter). It encodes the enzyme phytanoyl-CoA hydroxylase (PAHX), which is important for the peroxisome function. Mutations in the *PEX7* gene account for a minority of patients with ARD (10%) [8,10]. This gene is located at chromosome 6 (p22-q24). It encodes the peroxisomal type 2 targeting signal receptor, which imports enzymes to the peroxisome [5] by recognition of a bipartite protein complex consisting of receptor PEX7 and a co-receptor. Cargo-loaded receptor complexes are translocated across the peroxisomal membrane by a transient pore and inside peroxisomes, and then cargo proteins are released [11].

We report on an adult patient with Adult Refsum disease in Puerto Rico. Studies of patients with retinitis pigmentosa in Puerto Rico were approved by the local Institutional Review Board (Protocol: #2290034668R001).

## Case presentation

**Figure 1:**
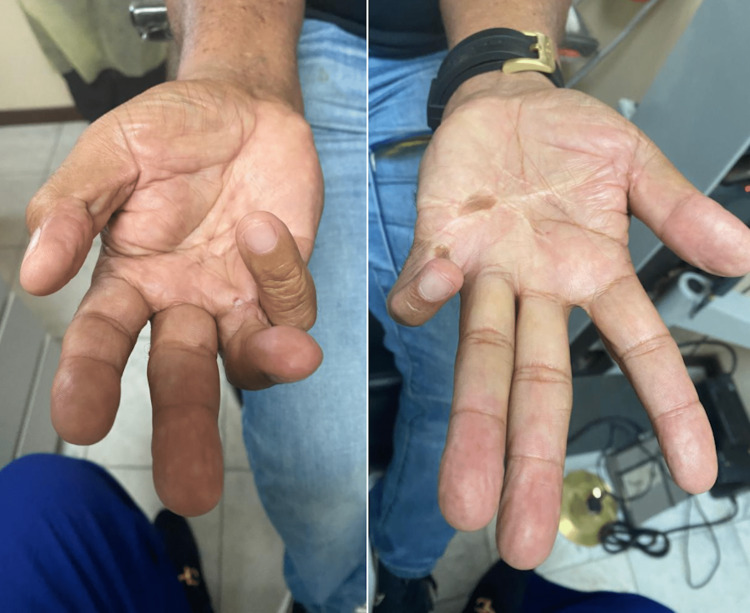
Evidence of surgical scars in both hands associated to peripheral neuropathy as part of the disease.

**Figure 2:**
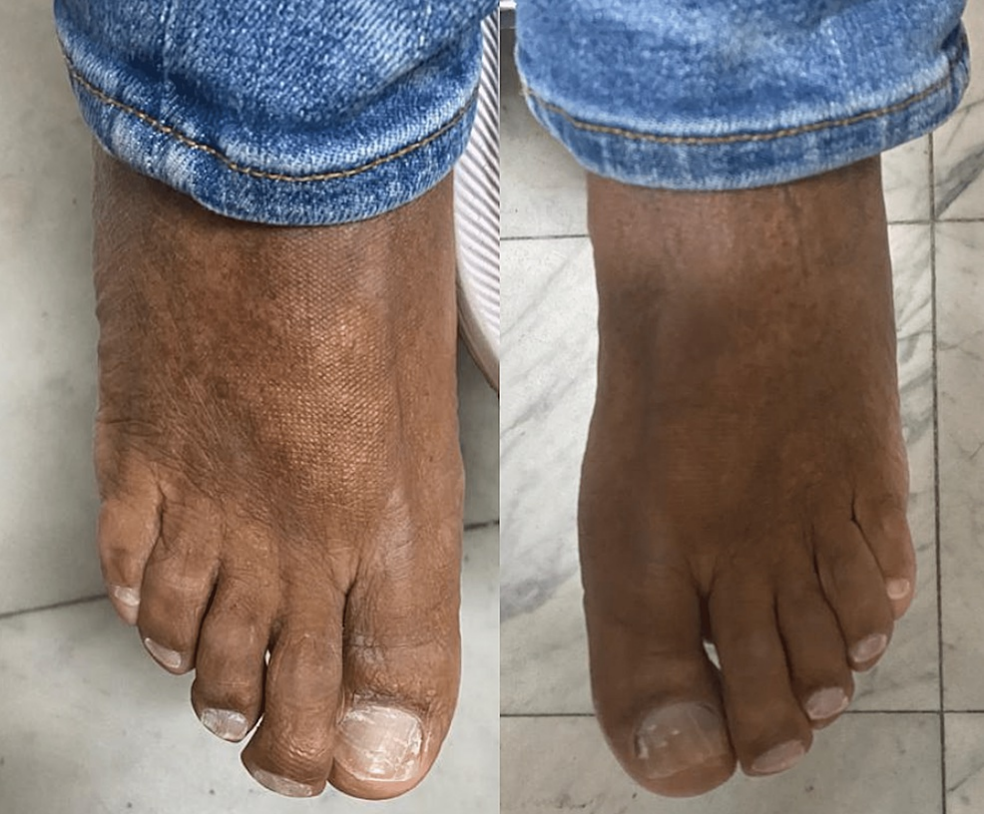
Evidence of length disproportion of the fourth and fifth toe of the right foot.

A 60-year-old male patient had a chief complaint of progressive nyctalopia. He had undergone cataract surgery in both eyes. The patient had undergone hand surgery due to chronic pain in both hands, as depicted in Figure 1. His fourth and fifth metatarsal bones were shortened, as shown in Figure 2.

The patient underwent a comprehensive ophthalmic evaluation by at least one of the authors (NIE). The best Corrected Visual acuity was 0.18 and 1.3 (log Mar) in the right and left eye, respectively. Refraction was +0.75 +0.50 x 10˚ and +0.75 +0.72 x 25˚ in the right and left eye, respectively. The patient had pseudophakia in both eyes. Upon fundus examination, the patient had pale optic nerves, arteriolar attenuation, paravenous retinal pigment epithelium (RPE) hyperplasia, a “salt-and-pepper” appearance surrounding the macula, and mid-peripheral bony spicules.

**Figure 3:**
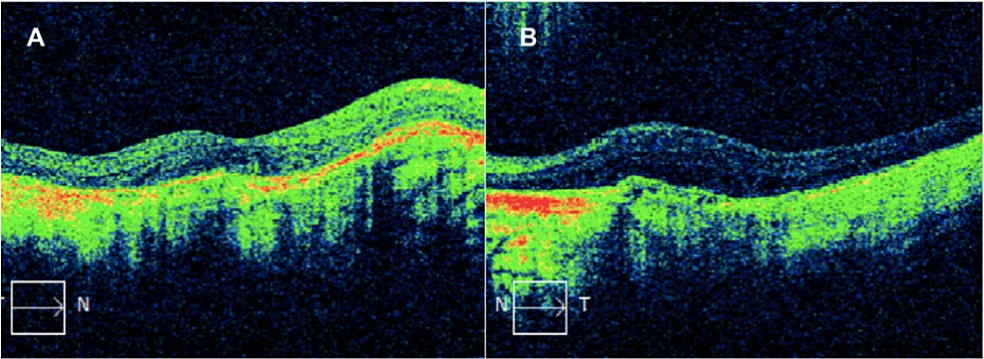
Optical coherence tomography (OCT) scan showing loss of foveal pit and several layers of the retina bilaterally.

Upon macular optical coherence tomography (OCT) (Carl Zeiss Meditec, Inc., Dublin, CA, USA), the patient had a macular thickness of 254 and 188 micrometers in the right (OD) and left eye (OS), respectively. Total macular volume was 9.1 mm³ and 6.8 mm³ in the OD and OS, respectively. Figure 3 portrays the OCT imaging, which depicts the loss of the foveal pit and several retina layers. Volume and thickness are decreased in both eyes. However, the findings are worse in the left eye.

Upon Visual Field testing (30-2 Carl Zeiss Meditec, Inc.), the patient had a mean deviation of -30.51 dB (p<0.5%) and -30.74 dB (p<0.5%) OD and OS, respectively. Pattern Standard Deviation was +4.43 dB (p<0.5%) and +5.04 dB (p<0.5%).

Full-field electroretinogram (ERG) (LKC Technologies, Inc., Gaithersburg, MD, USA) results showed scotopic ERG responses were non-recordable, while the photopic ERG responses were diminished OU, consistent with progressive rod-cone dystrophy.

Gene sequencing and deletion/duplication analysis using next-generation sequencing (NGS) (Invitae Corporation, San Francisco, CA, USA) were positive for a Variable of Uncertain Significance as a heterozygous mutation in the PHYH gene of the variant c.290G>A (p.Gly97Glu).

Audiologic studies showed a mild hearing loss from 4 to 6 KHz in the Right Ear. Mild to moderate hearing loss from 3 to 8 KHz at the left ear.

## Discussion

Previous studies have reported that patients with ARD have clinical findings compatible with retinitis pigmentosa [9]. Our patient had nyctalopia, retinal findings, and visual field results as part of retinitis pigmentosa. These findings are compatible with previous literature.

Cataracts have been reported in patients with retinitis pigmentosa [12]. Our patient had undergone cataract surgery in both eyes. This is compatible with previous studies.

Vámos et al. [13] have reported that patients with retinitis pigmentosa have decreased macular thickness. Our patient had decreased macular thickness in both eyes. Our findings are compatible with previous studies.

O'Neal and Luther [14] reported that patients with retinitis pigmentosa have increased macular volume that could be related to macular edema. Our patient had decreased macular volume in both eyes, worst in the left eye. The patient had no macular edema and thus did not have increased macular volume.

Previous studies have reported that patients with ARD have hearing loss as part of the disease [5, 15]. Our patient’s audiological evaluation showed mild hearing loss in the right ear and mild to moderate hearing loss in the left ear. This finding is compatible with the disease.

Two main genes have been associated with adult Refsum disease (i.e., *PHYH* and *PEX7*) [9, 16]. Gene sequencing and deletion/duplication analysis using next-generation sequencing (NGS) were positive for a Variable of Uncertain Significance as a heterozygous mutation in the PHYH gene of the variant c.290G>A (p.Gly97Glu). To our knowledge, this is the first report on Adult Refsum disease with this variant.

Limitations of the study include that there are very few patients with ARD since this is a rare disease. To our knowledge, this is the first report of adult Refsum disease in Puerto Rico.

## Conclusions

Clinical diagnosis of patients with deafblindness remains challenging. Adult Refsum disease must remain part of the differential diagnosis. Inheritance patterns in patients with ARD have been described. Physical examination, phytanic acid evaluation, and genetic studies may all contribute to reaching an ARD diagnosis. Prompt diagnosis may help co-manage and improve the prognosis of patients with the disease. Although genetic testing is an increasingly helpful tool to aid in diagnosing and managing these patients, technological shortcomings, such as Variants of Unknown Significance, incomplete testing, and unidentified related genes, should be considered.

Our patient, with a heterozygous mutation in the *PHYH* gene and a clinical ARD diagnosis, still has much to learn about inheritance, genetics, and the disease. Further studies of patients with ARD are needed in Puerto Rico.
